# AtaT Improves the Stability of Pore-Forming Protein EspB by Acetylating Lysine 206 to Enhance Strain Virulence

**DOI:** 10.3389/fmicb.2021.627141

**Published:** 2021-03-01

**Authors:** Zhili He, Tao Li, Jianxin Wang, Deyan Luo, Nianzhi Ning, Zhan Li, Fanghong Chen, Hui Wang

**Affiliations:** State Key Laboratory of Pathogens and Biosecurity, Beijing Institute of Microbiology and Epidemiology, Beijing, China

**Keywords:** Gcn5-related *N*-acetyltransferase, AtaT, enterohemorrhagic *Escherichia coli*, EspB, virulence

## Abstract

A novel type II toxin of toxin–antitoxin systems (TAs), Gcn5-related *N*-acetyltransferase (GNAT) family, was reported recently. GNAT toxins are mainly present in pathogenic species, but studies of their involvement in pathogenicity are rare. This study discovered that the GANT toxin AtaT in enterohemorrhagic *Escherichia coli* (EHEC) can significantly enhance strain pathogenicity. First, we detected the virulence of Δ*ataT* and Δ*ataR* in cell and animal models. In the absence of *ataT*, strains showed a lower adhesion number, and host cells presented weaker attaching and effacing lesions, inflammatory response, and pathological injury. Next, we screened the acetylation substrate of AtaT to understand the underlying mechanism. Results showed that *E. coli* pore-forming protein EspB, which acts as a translocon in type III secretion system (T3SS) in strains, can be acetylated specifically by AtaT. The acetylation of K206 in EspB increases protein stability and maintains the efficiency of effectors translocating into host cells to cause close adhesion and tissue damage.

## Introduction

Toxin–antitoxin systems (TAs) are widely present on prokaryote plasmids and chromosomes (Gerdes et al., 2005). These systems consist of two co-expression genes, encoding stable toxin and sensitive antitoxin. TAs were initially discovered as plasmid maintenance modules (Ogura and Hiraga, 1983; Gerdes et al., 1986; Bravo et al., 1987). Unstable antitoxins in plasmid-free cells cannot be supplemented, leaving the toxin free to eliminate or arrest the growth of these cells, which results in the maintenance of plasmids in the bacterial population. The advent of genome sequencing has further exposed the abundant TAs encoded on prokaryotes’ chromosomes (Van Melderen and Saavedra De Bast, 2009; Riffaud et al., 2020). Chromosome TA modules do not maintain plasmid stability. Their functions are associated with different metabolic processes and growth controls to cope with adverse environments (Yamaguchi and Inouye, 2011; Lobato-Marquez et al., 2016; Rocker and Meinhart, 2016; Harms et al., 2018; Paul et al., 2019; Walling and Butler, 2019). However, there are heated debates about the participation of chromosome TAs in environmental stress (Tsilibaris et al., 2007; Goormaghtigh et al., 2018a,b; Holden and Errington, 2018; Fraikin et al., 2020), and their role in organisms is more complicated and confusing than ever.

There are six different types of TAs according to the mechanism of toxin and antitoxin interaction, of which type II TAs research is the most in-depth. In recent years, scientists have discovered a new type II toxin that belongs to the Gcn5-related *N*-acetyltransferase (GNAT) (Cheverton et al., 2016; Jurenas et al., 2017; Qian et al., 2018, 2019; Wilcox et al., 2018). GNAT toxin blocks protein translation by acetylating the amino group of charged tRNAs, thus preventing tRNA from participating in peptidyl ribosomal transferase (Yeo, 2018). Reports suggest that GNAT toxins are mainly present in pathogenic species. Most GNAT toxins are distributed in the genomes of *Salmonella enterica* and *Klebsiella pneumoniae* (Xie et al., 2018); other major species carrying GNAT toxins are *Escherichia coli* and *Mycobacterium tuberculosis* (Qian et al., 2018). To date, the functions of several GNAT toxins in pathogenic species have been analyzed. KacT toxin contributes to antibiotic tolerance in *K. pneumoniae* (Qian et al., 2018); the expression of TacT in *Salmonella typhimurium* promotes the strain persistence in macrophages (Cheverton et al., 2016; Rycroft et al., 2018); and GmvT in *Shigella* stabilizes pathogenicity island-harboring pINV plasmid (McVicker and Tang, 2016). However, the function of GNAT toxins in other species is still largely unknown.

Enterohemorrhagic *E. coli* (EHEC) is a pathogenic Gram-negative bacterium. It can cause severe hemorrhagic colitis and hemolytic uremic syndrome in infected human bodies. It is also highly contagious and can lead to pandemic outbreaks. Epidemiological control is a challenge because it has a low infectious dose and sophisticated pathogenic mechanisms (Cameron et al., 2018; Lang et al., 2018). AtaT, a recently discovered GNAT toxin in *E. coli* O157:H7, uses acetyl-coenzyme A to block translation initiation by specifically acetylating tRNA^fMet^ (Jurenas et al., 2017). The antitoxin AtaR can prevent it from forming an active dimer to neutralize its toxicity (Yashiro et al., 2019). Jurënas and his colleagues explored the function of this TAs and concluded that the AtaRT system might be involved in anti-addiction and that AtaT is unlikely to participate in persistence (Van Melderen et al., 2018). A quick BLAST search shows that AtaRT is also widely distributed in *E. coli* O157:H7 Sakai, Xuzhou21, and many other pathogenic strains including *E. coli* O55:H7 RM12579, CB9615, *E. coli* APEC O78, and *E. coli* NA114. As a GNAT toxin mainly distributed in pathogenic species, it is unclear whether *ataT* is involved in strains’ pathogenic processes.

In this study, we revealed that GNAT toxin AtaT enhances the virulence of strains by maintaining the stability of pore-forming protein EspB through acetylation.

## Materials and Methods

### Ethics Statement

This study was carried out according to the recommendations set out in the Guide for the Beijing Institute of Microbiology and Epidemiology Animal Care and Use Committee (2016-05-11-05). The protocol was approved by the Institutional Ethics Review Committee of Beijing Institute of Microbiology and Epidemiology, China. Mice were purchased from Vital River Laboratory Animal Technology, Beijing, China (permit number: 2016-0006). The female BALB/c mice (14–16 g) were maintained on either a regular diet (standard mice feed and 12 h for light/dark alternate). All animal work was carried out strictly under the approved guidelines, and all efforts were made to minimize suffering.

### Bacterial Strains and Plasmids

All of the strains and plasmids used in this study are listed in Supplementary Table 1. Bacteria were grown with shaking at 37°C in lysogenic broth (LB) culture or Dulbecco’s modified Eagle’s medium (DMEM) media. Ampicillin (100 μg/ml), chloramphenicol (25 μg/ml), kanamycin (50 μg/ml), and apramycin (60 μg/ml) were used in this study. Wherever indicated, 0.2% L-arabinose or 0.1 mM of isopropyl β-D-1-thiogalactopyranoside (IPTG) was used as an inducer.

### Construction of Mutant Strain

Mutant strains of EHEC were constructed using the λ Red recombineering gene deletion technology. The kanamycin-resistant gene kan (flanked by flippase recognition target FRT sites) flanked by homologous sequences of *ataT* was amplified by PCR. PCR fragments were analyzed by agarose gel electrophoresis on 1% (wt/vol) agarose gels for 30 min. PCR fragments were purified using the PCR product purified kit (Transgen Biotech). P8 strain (EHEC harboring plasmid pKD46) was grown to an optical density at 600 nm (OD600) of ∼0.6 at 30°C and washed three times with water and glycerol [10% (vol/vol)]. Two hundred nanograms of PCR fragments was electroporated into P8 (Bio-Rad MicroPulser Electroporator). Cells were then recovered in 0.7 ml of LB for 1 h, plated on LB plus agar (kanamycin), and incubated overnight at 30°C. The Δ*ataT* strain carrying the kana-resistance gene (Δ*ataT*:kana) was obtained. Δ*ataT*:kana was streaked repeatedly on antibiotic plates at 42°C to cure the strains of plasmid pKD46. Then the plasmid pFLP2 was electroporated into Δ*ataT*:kana to remove the kanamycin-resistant gene. pFLP2 was cured via culture in LB with 6% sucrose (w/v). The Δ*ataR* strain was engineered as follows.

### Cellular Infection

The day before infection, approximately 1 × 10^5^ of HT-29 (human colon cancer cells) were seeded in DMEM (10% fetal bovine serum, #10099141C, Gibco) on six-well plates or coverslips without antibiotics. Overnight culture of strains EHEC wild-type (WT), Δ*ataT*, and Δ*ataR* were transferred into fresh DMEM medium at a ratio of 1:1,000. When OD600 nm = 0.6, cells were infected at multiplicity of infection (MOI) = 10 for different periods of time. Phosphate-buffered saline (PBS)-treated cells were used as a control. Next, we processed the different batches of cells in the following steps: (1) we removed supernatants and treated the cells with 0.25% trypsin (#25200072, Gibco), and the bacteria adhering to the cells were collected. Total RNA was isolated (#ER501-01, Trans, Beijing, China), and cDNA was generated (#AT341-02, Trans, Beijing, China) by reverse transcription using RNA as a template. The levels of mRNA were analyzed by quantitative real-time PCR (#AQ131-03, Trans, Beijing, China). (2) We removed supernatants, and the cells were washed three times with PBS. After being treated with 0.25% trypsin for 2 min and 0.025% Triton X-100 for 10 min, the cell suspension per well was fixed to a volume of 1 ml by PBS. The gradient-diluted cell lysates were inoculated on agar plates, and bacterial colonies were counted. (3) After re-culturing in DMEM containing antibiotics for another 6–8 h, the level of IL-8 in the supernatant was detected by ELISA (#EHC008.96, NeoBioscience, Beijing, China). (4) HT-29 cells on the coverslips were fixed and stained with antibody to *E. coli* O157 (#ab156617, Abcam), Alexa Fluor 488 goat anti-mouse IgG (#A-11029, Invitrogen), Alexa Fluor 647 phalloidin (#A30107, Invitrogen), and DAPI (#C0065, Solarbio, Beijing, China); and the slices were subjected to fluorescence microscopy (Zeiss LSM880).

### Mice Infection

Fifteen female BALB/c mice (pre-treated with 5 g/L of streptomycin for 3 days) per group were orally gavaged with EHEC WT, Δ*ataT*, or Δ*ataR* [1 × 10^9^ colony-forming units (CFU)]. At the same time, PBS was used as a negative control. Feces from five mice were randomly taken from each group in indicated days, and the bacterial shedding was counted on Sorbitol-MacConkey agar plates until the 23rd day. On the fourth day of infection, blood was collected from five randomly selected mice in each group. We detected mice serum keratinocyte-derived cytokine (KC) concentration using a mouse KC kit (EMC104.96, NeoBioscience, China). We isolated the colon; then fixed, sliced, and stained it; and analyzed pathological lesions.

### Protein Expression and Purification

*Escherichia coli* BL21(DE3) carrying the pETDuet-1-EspB plasmid, expressing EspB (His)_6_, was grown to the logarithmic phase at 37°C. IPTG was added to make the final concentration 0.1 mM and further cultured for 16 h at 19°C. Strains were centrifugated at 4°C and disrupted by ultrasonic method. After centrifugation, the sediment was resuspended in urea buffer (8 M of urea, 100 mM of Tris–HCl, pH 8.0) overnight and renatured by step-by-step dialysis. AtaT (His)_6_ was purified from *E. coli* BL21(DE3) harboring pETDuet-1-AtaRT. The complex of AtaR-AtaT (His)_6_ was captured by Ni-NTA resin for the first time. After dissociation of AtaR from the complex in 5 M of guanidine-HCl, AtaT (His)_6_ was recaptured by Ni-NTA resin and renatured. Fractions containing purified AtaT (His)_6_ or EspB (His)_6_ were selected based on sodium dodecyl sulfate–polyacrylamide gel electrophoresis (SDS–PAGE) analysis.

### Acetylation Assays *in vivo* and *in vitro*

The acetylation *in vivo* assay steps were as follows. Vectors pETDut1-*espB*(his)_6_ and pBAD33-*ataT* were co-expressed in *E. coli* BL21 (DE3), while pETDut1-*espB* was expressed alone as a control. IPTG or L-arabinose were added at 1 h to induce the expression of EspB or AtaT, and the same level of EspB (samples of co-expressed strains were condensed about five times) was collected at 6 h. The modification levels of EspB were detected by acetylated antibody (α-Acetyl) (#9441, CST). Histidine antibody (anti-His) (#12698, CST) was used as a control. Other adhesion proteins [EspA(his)_6_, LpfA(his)_6_, Tccp(his)_6_, Intimin(his)_6_, and Tir(his)_6_] followed the same experimental procedures to detect the level of acetylation.

The *in vitro* acetylation reaction was performed at 37°C for 6 h by adding 10 μg of AtaT, 4 μg of EspB, and 0.2 mM of acetyl-CoA in a volume of 50 μl to produce the EspB-Ac (Ren et al., 2016), while EspB incubated alone as a control.

### Degradation of EspB *in vitro*

Dulbecco’s modified Eagle’s medium with 1 g/L glucose was inoculated 1:100 with overnight cultures of EHEC allowed to grow in 5% CO_2_ at 37°C. After 16 h of growth, the supernatant was collected and filtered through a 0.22-μm-pore-size filter unit (Millipore) to concentrate 50-fold to produce the endogenous protease of EHEC (Cameron et al., 2018). Five hundred nanogram of EspB or EspB-Ac (from acetylation assays *in vitro*) was exposed to 150 μl of concentrated protease and incubated at 37°C for 5 h. The same volume of mixtures was collected every hour, and protein levels with histidine antibody (anti-His) detected.

### Degradation of EspB *in vivo*

The stability of EspB (His)_6_ and its derivative mutants was detected via *in vivo* degradation experiments (Sang et al., 2016). Plasmid harboring *espB*(his)_6_ or its derivatives were induced by 0.1 mM of IPTG for 60 min. Translation was blocked with 100 μg/ml of spectinomycin, and samples were collected every 10 min. The EspB levels were detected with histidine antibody (anti-His), while Dnak levels were detected as a control.

### Translocation Assay

HeLa cells were infected with EHEC WT and mutants (harboring the Tir-TEM1 vector, MOI = 10). After 30 min, IPTG was added and incubated for another 4 h. Cell monolayers were washed with Hank’s balanced salt solution (HBSS) three times and covered with CCF2/AM solution (#K1023, Invitrogen) for 1 h in darkness at room temperature. The CCF2/AM solution was washed off with HBSS, and cells were observed under a confocal fluorescence microscope (Dichroic mirror, Zeiss LSM880). After being excited by 405 nm, the ratio of blue fluorescence (460 nm) to green fluorescence (530 nm) was collected.

## Results

### AtaT Enhanced the Adhesion of Enterohemorrhagic *Escherichia coli* and Inflammatory Response of Host Cells

**FIGURE 1:**
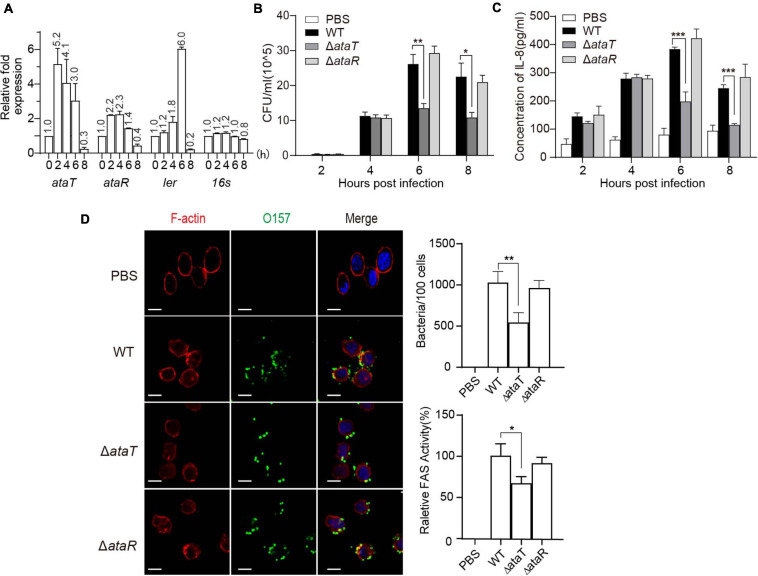
AtaT promotes the adhesion of enterohemorrhagic *Escherichia coli* and the inflammatory responses of host cells. **(A)** *ataRT* mRNA level in WT strains during infection. **(B)** AtaT promotes the adhesion ability of EHEC in HT-29 cells. EHEC WT, Δ*ataT*, and Δ*ataR* infected HT-29 cells at MOI = 10 for different periods of time. The gradient diluted cell lysates were inoculated on agar plates, and bacterial colonies were counted to calculate adhesion efficiency. **(C)** AtaT enhanced the secretion of IL-8 in cells. WT, Δ*ataT*, and Δ*ataR* strains infected HT-29 cells for hours. The level of IL-8 in the culture supernatant was detected by ELISA after cells were re-cultured in DMEM containing antibiotics for another 6–8 h, and PBS-treated cells served as controls. **(D)** WT, Δ*ataT*, and Δ*ataR* strains infected HT-29 cells for 6 h. Cells were stained with anti-O157 (green), DAPI (blue), and phalloidin (pink), while white bars represent 10 μm. Quantification of the adhesion strains results is shown at the top. The degree of A/E was quantified by the ratio of fluorescence intensity (actin staining/bacterial staining) (shown at the bottom). All of the experiments shown were repeated at least two times, and error bars represent ± SEM. *P < 0.05, **P < 0.01, ***P < 0.001 compared with EHEC WT. DAPI, 4′,6-diamidino-2-phenylindole; WT, wild-type; ELISA, enzyme-linked immunosorbent assay; IL-8, interleukin 8; EHEC, enterohemorrhagic *Escherichia coli*; MOI, multiplicity of infection; DMEM, Dulbecco’s modified Eagle’s medium; PBS, phosphate-buffered saline; A/E, attaching and effacing.

Since *ataRT* is mainly distributed in pathogenic intestinal bacteria, we first explored the relationship between AtaRT and pathogenicity. We used the host cells of EHEC, HT-29 cells, infected with exponential-phase EHEC for hours, and the adherent bacteria were collected while EHEC was grown without cells as a control. Isolated total RNA and real-time qPCR data were analyzed. As shown in Figure 1A, at post-infection 2–6 h, *ataT* and *ataR* were upregulated, and their expression decreased at 8 h. This was consistent with *ler*, which encodes Ler protein (LEE-encoded regulator) and is one of the essential regulatory molecules during EHEC adhesion (Alsharif et al., 2015).

Then, we assessed several pathogenicity indicators in EHEC, including Shiga toxin production ability and adhesion ability. The growth curves of the gene-deleted mutants Δ*ataT* and Δ*ataR* are shown in Supplementary Figure 1B. The Δ*ataT* growth conditions were consistent with EHEC WT, while Δ*ataR* exhibited growth inhibition because the repression of the toxin gene *ataT* was released (Supplementary Figure 1C). We next detected the levels of Shiga toxin produced by these strains when infecting HT-29 cells for 6 h, and no significant difference was observed (Supplementary Figure 2). However, in the adhesion test experiment, we found an interesting phenomenon. The bacterial adhesion number of Δ*ataT* was reduced by 50% compared with WT at 6 and 8 h, and the adhesion of Δ*ataR* was consistent with WT (Figure 1B). This means that the toxin may be involved in the adhesion of the strain. Cytokines are responsible for regulating the inflammatory response. More bacterial adhesion will lead to more severe inflammation. We therefore collected the supernatants of infected cells to detect the levels of cell inflammatory factor IL-8. As expected, WT and Δ*ataR* groups possessed higher inflammatory response than the Δ*ataT*-infected group at 6 and 8 h (Figure 1C). Relative fluorescence actin staining (FAS) activity (Luo and Donnenberg, 2006) under the microscope also showed that the attaching and effacing (A/E) lesions of cells in the Δ*ataT*-infected group were significantly less than those in the WT and Δ*ataR* groups (Figure 1D). All of the results demonstrated that type II toxins AtaT can enhance EHEC’s adhesion ability and cause several inflammatory responses.

### The ΔataT Attenuated Bacterial Localization and Inflammation in the Colon

**FIGURE 2:**
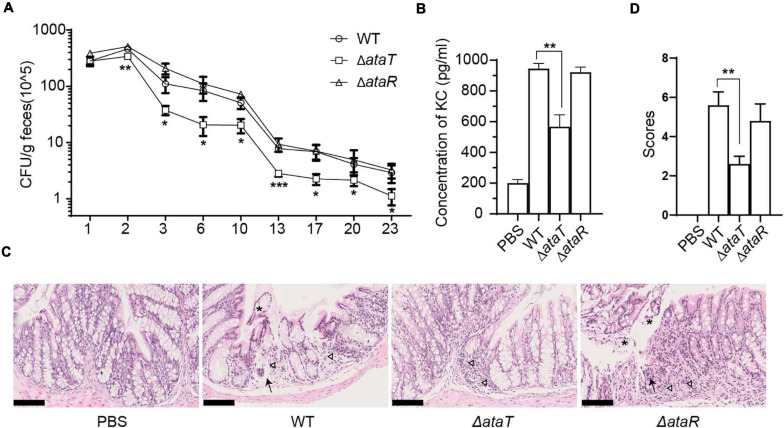
AtaT increases colonization of EHEC and causes severe inflammation in the colon. **(A)** Magnitude of fecal shedding of different strains. WT, Δ*ataT*, and Δ*ataR* strains infected streptomycin-treated BALB/c mice for up to 23 days. Feces suspensions were plated on Sorbitol-MacConkey agar plates, and bacterial counts are plotted as the average CFU per gram of feces. **(B)** AtaT increases the level of serum KC levels. ELISA detected the levels of KC on the fourth day and PBS-gavaged mice served as controls. **(C)** AtaT increases colon injury in infected mice. The colons of mice infected for 4 days in each group were sectioned, fixed, and stained. Inflammatory cells (triangle), exfoliated epithelium (asterisk), and acinar atrophy with local edema (arrow) were marked, and bars represent 100 μm. **(D)** The colon injury severity score. The degree of colonic pathological injury ranged from the slightest to the most severe, with a score of 0–10. Three to five fields of vision were selected for each slide. Error bars represent ± SEM. *P < 0.05, **P < 0.01, ***P < 0.001 compared with EHEC WT. All of the experiments shown were repeated at least two times. WT, wild-type; ELISA, enzyme-linked immunosorbent assay; CFU, colony-forming units; KC, keratinocyte-derived cytokine; EHEC, enterohemorrhagic *Escherichia coli*; PBS, phosphate-buffered saline.

As a molecule that aggravates cell infection, we wanted to know the role of AtaRT in mouse models. BALB/c female mice were randomly divided into four groups with 15 mice in each group. They were given intragastric administration of 1.0 × 10^9^ CFU of WT, Δ*ataT*, or Δ*ataR*. We detected the amount of strain shedding in the feces and tested the colonization of different strains. Feces suspensions were plated on Sorbitol-MacConkey agar plates, and bacterial counts were plotted as the average CFU per gram of feces. We observed that the strain excretion in group Δ*ataT* was markedly lower than in the other two groups from days 3 to 23 (Figure 2A). This test confirmed that AtaT maintained the strong intestinal colonization ability of the strain. We detected the KC, a functional homolog of human IL-8 (Li et al., 2017), levels in the serum on the fourth day. As depicted in Figure 2B, compared with the WT-infected group, deletion of *ataT* significantly decreased KC levels by approximately 50%, while Δ*ataR* could replenish KC levels. We also took out the colon and fixed the section to evaluate the pathological lesions on this day. There were distinct pathology lesions in WT or ΔataR-infected mice, such as the intestinal cavity showing shedding epithelium, infiltration of inflammatory cells in the acinus lamina propria acinar absence atrophy, and local edema. Only mild inflammation was observed in Δ*ataT*-infected mice (Figure 2C). At the same time, we set the scoring criteria and scored the slices. The colon injury severity scores clearly showed that colon inflammation of Δ*ataT*-infected mice was much milder (*P* < 0.01), but that the colon remained severely inflamed after infection with EHEC WT or Δ*ataR* (Figure 2D), indicating that less Δ*ataT* colonized colon and caused slighter pathological damage.

### AtaT Promotes the Stability of EspB by Acetylation to Enhance the Translocation of Effectors Into Host Cells

**FIGURE 3:**
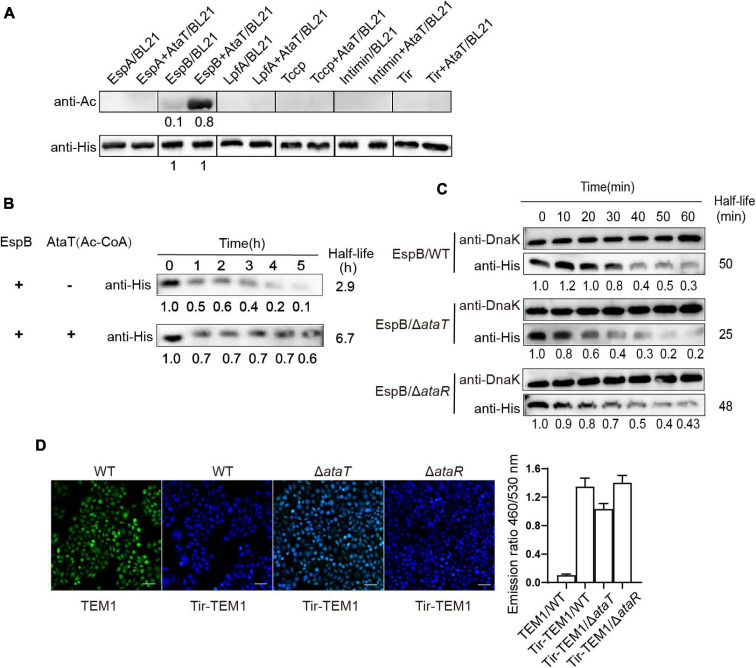
EspB promotes its stability through acetylation by AtaT to keep Tir translocate into host cells. **(A)** EspB is the AtaT-specific acetylation modification target *in vivo*. Detection of the acetylation level of adhesion proteins [EspA(his)_6_, EspB(his)_6_, LpfA(his)_6_, Tccp(his)_6_, Intimin(his)_6_, and Tir(his)_6_], which are expressed alone or co-expressed with AtaT in *Escherichia coli* strain BL21. Acetylation levels were detected by the anti-acetyl lysine antibody (anti-Ac) and the anti-His antibody (anti-His) was used as a control. **(B)** Acetylation of EspB enhances protein stability *in vitro*. Purified 6xHis-tagged EspB was exposed to T3SS enzyme with or without purified AtaT and Ac-CoA for hours. Protein levels were detected by anti-His antibody. **(C)** Stability of EspB in EHEC WT, Δ*ataT*, and Δ*ataR* strain. Strains harboring EspB expression plasmid was induced by IPTG for 1 h. Spectinomycin was used to terminate protein synthesis at different points, and strains were collected. The level of EspB was detected by anti-His antibody, while Dnak levels were detected as controls. The gray value calculated the half-life. **(D)** Acetylation of EspB enhances Tir into host cells via T3SS. HeLa cells were infected with wild-type EHEC or mutants containing Tir-TEM1 vectors. At 6 h of infection, cells were incubated with CCF2/AM1 dye. The β-lactamase activity in HeLa cells was detected by measuring the CCF2/AM substrate’s cleavage and presented as the emission ratio of blue/green fluorescence (460/530 nm). White bars indicate 50 μm, data are represented as mean ± SEM, and experiments shown were repeated at least two times. T3SS, type III secretion system; IPTG, isopropyl β-D-1-thiogalactopyranoside; EHEC, enterohemorrhagic *Escherichia coli*.

We then explored the molecular mechanism of AtaT increasing EHEC colonization. AtaT has been identified as a member of the acetyltransferase family. We co-expressed various adhesion proteins with AtaT or without and detected the acetylation levels of these proteins. As AtaT affects bacterial growth (Supplementary Figure 3), samples of co-expression groups needed to be concentrated about fivefold to reach the same protein level as the controls. As shown in Figure 3A, the acetylation level of EspB with AtaT co-expression increased eightfold compared with expression alone. However, other adhesion proteins, such as Tir and Intimin, could not be acetylated, regardless of whether AtaT was present. These results showed that EspB is the specific substrate of AtaT; this may explain why AtaT can maintain the strain intestinal colonization. In addition, we performed degradation systems for EspB *in vitro* and *in vivo*. First, purified EspB and EspB-Ac (treated with AtaT and acetyl-CoA) were exposed to endogenous protease EspP (*E. coli*-secreted protein P) from EHEC (Cameron et al., 2018). It was observed the half-life of purified EspB increased 2.3-fold after being treated with AtaT *in vitro* (Figure 3B). Subsequently, compared with that of WT, the half-life of EspB in Δ*ataT* was shortened to nearly 50%. The stability of EspB can be restored by replenishing *ataT* in the background of Δ*ataR in vivo* (Figure 3C). The above results suggested that AtaT maintains the stability of EspB *in vivo* and *in vitro*.

EspB is a translocon protein of the type III secretion system (T3SS) and forms the T3SS pore in host cells. As an adhesion protein, Tir is the first effector to be injected into host cells (Mills et al., 2013). If the stability of EspB changes, the translocation of Tir through T3SS is also affected. Therefore, we tested Tir translocation efficiency in mutants and WT strains by using the TEM-1-β-lactamase reporter. Strains harboring the Tir-TEM1 or TEM1 vector infected HeLa cells for 6 h. The relative translocation efficiency of TEM-1 was represented by the emission ratio of 460/530 nm. It was shown that when *ataT* was absent, the emission ratio of 460/530 nm (blue/green) was reduced to about 77% compared with the WT or Δ*ataR* groups (Figure 3D). Therefore, we concluded that AtaT acetylates EspB to maintain its stability, further enhancing the translocation of adhesion protein Tir into host cells.

### Acetylation of K206 Enhances EspB Stability and Modulates the Virulence of Enterohemorrhagic *Escherichia coli*

**FIGURE 4:**
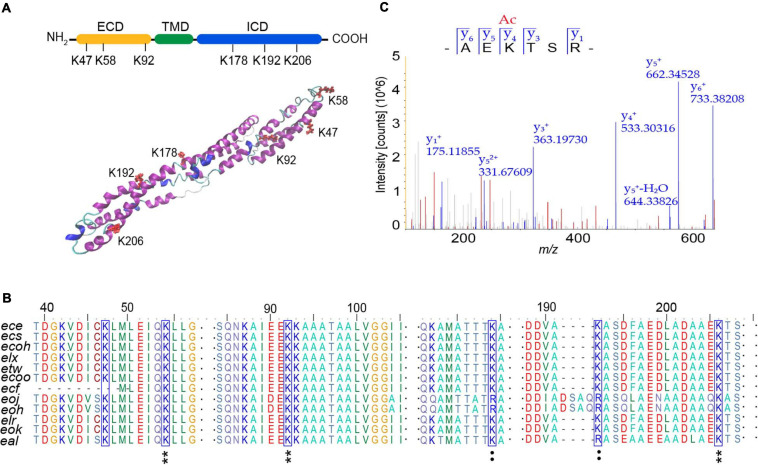
Analysis of acetylation sites modified by AtaT in EspB. **(A)** Acetylation sites in domain architecture (top) and predicted tertiary structure of EspB (bottom). Structure prediction was performed with I-TASSER. ECD, extracellular domain, yellow. ICD, intracellular domain, green. TMD, transmembrane domain, blue. Lysine residues detected to be acylated (K47, K58, K92, K178, K192, and K206) are highlighted in red. **(B)** Conservation analysis of EspB lysine residues (blue box) through sequence alignment. Star, conserved; colon, conservative change; and the result was analyzed by BioEdit. **(C)** MS/MS spectrum of EspB K206 acetylated (K206Ac) peptide detected from EspB co-expression with AtaT in *Escherichia coli* BL21(DE)3. MS, mass spectroscopy.

In order to determine the modification sites of AtaT acetylated EspB, two EspB protein samples were analyzed by mass spectrometry. One was 6xHis-EspB expressed from strain *E. coli* BL21(DE3) carrying the vector pET-EspB(his)_6_, and the other one was the above vector co-expressed with vector pBAD-AtaT. Of the 23 lysine residues in EspB, we identified six lysines (K47, K58, K92, K178, K192, and K206) by liquid chromatography–tandem mass spectroscopy (LC-MS/MS) analysis (Supplementary Table 2). We used I-TASTER to predict the tertiary structure of EspB. We found that all six acylated lysine residues were located at the exposed sites; K47, K58, and K92 were in the extracellular domain; and K178, K192, and K206 were in the intracellular domain (Figure 4A). Among them, K206 was the most significant acetylation site when comparing the ratio of acetylated peptides with unmodified peptides (Figure 4C and Supplementary Table 2).

**FIGURE 5:**
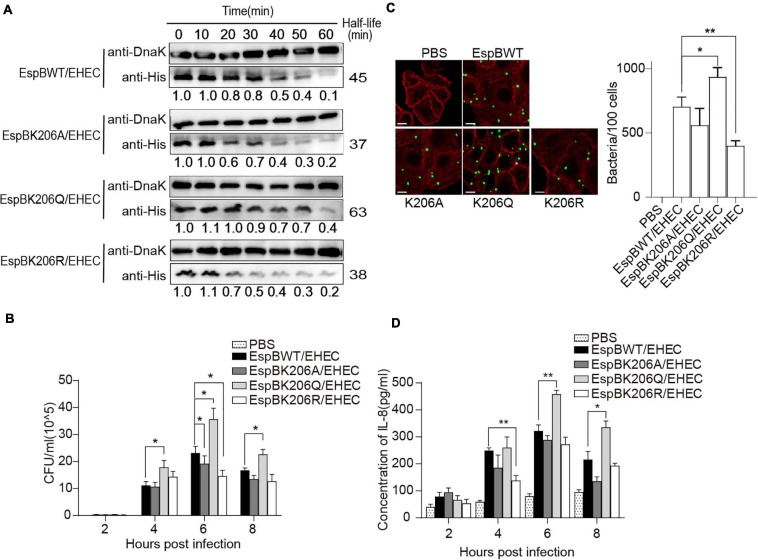
Acetylation of EspB K206 enhances protein stability and promote the virulence of strains. **(A)** Stabilities of EspB WT, K206Q, K206R, and K206A in EHEC. Strains harboring EspB expression plasmid were induced by IPTG for 1 h. Spectinomycin was used to terminate protein synthesis at different points and strains were collected. Levels of EspB were determined by anti-His antibody, while Dnak levels were detected as controls. The half-lives were calculated by the gray value. **(B)** EspB K206Q enhances EHEC adhesion ability to host cells. HT-29 cells were infected with EHEC harboring EspB wild-type or its derivatives at an MOI of 10. The gradient diluted cell lysates were inoculated on agar plates, and bacterial colonies were counted to calculate adhesion efficiency. **(C)** The number of bacteria attached after cells were infected for 6 h was observed under the fluorescence microscope. **(D)** IL-8 expression levels of host cells in different infection hours were determined by ELISA. Error bars represent SEM from ≥2 independent experiments, *P < 0.05, **P < 0.01 compared with EspB WT/EHEC. WT, wild-type; IPTG, isopropyl β-D-1-thiogalactopyranoside; ELISA, enzyme-linked immunosorbent assay; IL-8, interleukin 8; EHEC, enterohemorrhagic *Escherichia coli*; MOI, multiplicity of infection.

To determine which sites are tightly associated with protein stability, these sites were mutated to alanine (A), glutamine (Q), or arginine (R), to mimic the non-acetylated, constitutively acetylated, and cannot be acetylated or deacetylated forms, respectively (Ren et al., 2016). We constructed these mutant expression vectors of EspB with six histidine tags at its C termini and tested these proteins’ stability in EHEC. The results showed that the half-life of K206Q was approximately 1.4-fold longer than that of EspB WT, while the stability of K206R and K206A was slightly reduced (Figure 5A). Changes in acetyl levels at other sites were either independent of stability or inconsistent with the change of EspB WT modified by AtaT (Supplementary Figure 4). Modification on these sites may affect the basic functions of EspB or be redundantly modified. Importantly, K206 is located in the predicted protein–protein interaction domains of EspB (Luo and Donnenberg, 2011) and is highly conserved in bacteria (Figure 4B). Therefore, we suspect that EspB maintains its stability mainly via acetylation at the K206 site by AtaT.

Furthermore, to examine the effect of acetylated K206 on EHEC virulence, we infected HeLa cells with EHEC harboring EspB WT or EspBK206 derivatives. As expected, K206Q or EspB WT carrying strains showed higher adhesion ability compared with K206R or K206A at 6 h of infection (Figures 5B,C), and there were also similar trends at other infection time points. The IL-8 levels also showed that host inflammatory responses in the K206R and K206A groups were less than those in the K206Q or EspB WT strains groups, which is consistent with the trend of adhesion (Figure 5D). The above results showed that EspB K206Q mimicking acetylation could significantly increase the virulence of EHEC.

## Discussion

Type II TA is the most deeply studied TA, and it is involved in a variety of cell physiological activities. Although the role of type II TAs in the formation of persister cells has been questioned in recent years (Goormaghtigh et al., 2018a; Pontes and Groisman, 2019; Rosendahl et al., 2020), other functions, such as growth diminution during stress, biofilm formation, and phage inhibition, are still widely recognized (Wen et al., 2014; Sauert et al., 2016; Hosseini et al., 2019; Jurënas and Van Melderen, 2020; Song and Wood, 2020). In this work, we demonstrated that the type II toxins AtaT is involved in the virulence of EHEC, including strains colonization, inducing severe pathological injury and host inflammatory response.

*ataRT*, locus Z4833–Z4832 in EHEC O157:H7 EDL933, which the toxin AtaT of the AtaRT, is a member of the GNAT family. Blast analyses online show that AtaRT is distributed in various pathogens, such as *Escherichia*, *Shigella*, *Klebsiella*, and *Salmonella* (Supplementary Figure 1A). The strain can also present a basal growth state in the absence of cognate antitoxin (Supplementary Figure 1B), suggesting that AtaT shows “weaker” toxicity. It was inferred that AtaT may have other non-toxic functions. In this study, *ataRT* (especially *ataT*) expression was upregulated during strain infection, and AtaT promotes EHEC virulence (Figures 1, 2). Although we do not know which infection signal triggered the TAs, these data provide strong evidence that the GNAT toxin AtaT is involved in regulating strains’ pathogenicity.

Adhesion plays a vital role in host colonization of strains. EHEC contains a battery of adhesion proteins, including intimin, Tir, Tccp, T3SS translocons (EspA, EspB, and EspD), and Lpf (long polar fimbria) (McWilliams and Torres, 2014). Many virulence proteins (effectors) are translocated into host cells via T3SS (Bohn et al., 2019), and EspB is the base element built on the host cell. Notably, AtaT can specifically acetylate EspB directly and promote its stability, so the adhesion proteins, such as Tir, can be efficiently transported into host cells, facilitating close attachment of the bacteria (Figure 3). Except for TacT, which can acetylate both TacA and tRNAs synchronously in *Salmonella*, no known GNAT toxin has been found to acetylate proteins (VanDrisse et al., 2017). Jurenas et al. (2017) used isotope-labeled (^14^C) Ac CoA to monitor the acetylation reaction catalyzed by the AtaT *in vitro* translation reaction. The product was dissolved on SDS–PAGE gels, and no signal was detected, so they suggested that either the target was not a protein or the acetylation was unstable in this condition. In our co-expression reaction in *E. coli* BL21(DE)3, we revealed an apparent increase in the degree of acetylation. This is the first report that AtaT can directly modify protein besides tRNA.

EspB maintains its stability mainly via the acetylation of K206. However, we do not know how the change in the acetylation state of EspB K206 regulates its stability. Recently, Eshun-Wilson et al. (2019) reported that acetylation improved α-tubulin stability and explained the structural mechanism. They showed that deacetylated K40 in an α1-monomer is close to the M-loop, supporting lateral interaction. When K40 is acetylated, it packs ∼10 Å closer to the globular domain, reducing the potential for intermonomer interactions. Data from another study (Song et al., 2019) showed that the deacetylation-mimic in K49 and K51 had a larger surface area than the acetylation-mimic. As a large surface area is often required for potent protein interactions, acetylation might decrease the binding affinity of LC3 family proteins to its potential interacting protein. As mentioned in the results, K206 is located in the predicted protein–protein interaction domains of EspB. We propose that the acetylation of K206 may alter the conformational landscape. Less surface area blocks EspP or other interacting proteins from approaching it, thereby hindering the degradation of acetylated EspB. Notably, AtaT and EspB are both widely present in EHEC and enteropathogenic *E. coli* (EPEC) (data not shown). In addition, the EspB modification site K206 is very conserved in the strain distribution (Figure 4), indicating that the model of AtaT modifying EspB is probably a comprehensive regulation. If we find molecules that can intervene or reverse K206 modification by AtaT, we may be able to deal with most of the pathogenic problems of EHEC and EPEC.

## Conclusion

**FIGURE 6:**
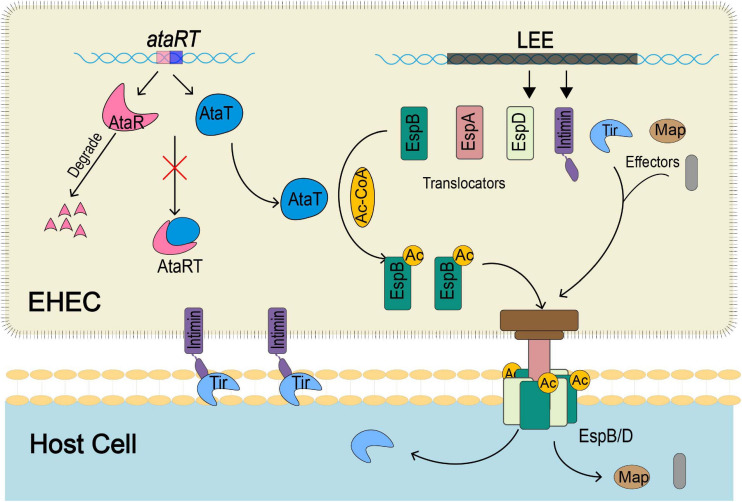
The schematic diagram of how EspB acetylated modification by AtaT enhances EHEC virulence. During host infection by strains, AtaT acetylates the EspBK206 site with acetyl-CoA, ensuring that EspB can be secreted extracellularly to form a stable T3SS base with EspD, enhancing early adhesion. Then Tir and other effectors transported into the host cell via T3SS efficiently cause tight attachment, host inflammatory responses, and tissue injury. T3SS, type III secretion system; EHEC, enterohemorrhagic *Escherichia coli*.

In summary, our study revealed that the GANT toxin AtaT could enhance the virulence of strains and explained the mechanism (Figure 6). *ataRT* genes upregulate during infection, and AtaT acetylates EspB with acetyl-CoA. The modified EspB is secreted extracellularly, forms a channel complex with EspD, and then stably embedded in the host cell membrane. Then effectors such as Tir are transported into the host cell efficiently, where they cause tight attachment, host inflammatory responses, and tissue injury.

## Data Availability Statement

The raw data supporting the conclusions of this article will be made available by the authors, without undue reservation.

## Ethics Statement

The animal study was reviewed and approved by the Institutional Ethics Review Committee of Beijing Institute of Microbiology and Epidemiology, China.

## Author Contributions

ZH performed the experiments, analyzed the data, and wrote the manuscript. TL, JW, ZL, DL, and NN analyzed the data. FC and ZL performed the experiments. HW designed this study, analyzed the data, wrote the manuscript, and supervised the project. All authors discussed the results and commented on the manuscript.

## Conflict of Interest

The authors declare that the research was conducted in the absence of any commercial or financial relationships that could be construed as a potential conflict of interest.

## References

[B1] Alsharif G. Ahmad S. Islam M. S. Shah R. Busby S. J. Krachler A. M. (2015). Host attachment and fluid shear are integrated into a mechanical signal regulating virulence in *Escherichia coli* O157:H7. *Proc. Natl. Acad. Sci. U.S.A.* 112 5503–5508. 10.1073/pnas.1422986112 25870295 PMC4418854

[B2] Bohn E. Sonnabend M. Klein K. Autenrieth I. B. (2019). Bacterial adhesion and host cell factors leading to effector protein injection by type III secretion system. *Int. J. Med. Microbiol.* 309 344–350. 10.1016/j.ijmm.2019.05.008 31178419

[B3] Bravo A. de Torrontegui G. Díaz R. (1987). Identification of components of a new stability system of plasmid R1, ParD, that is close to the origin of replication of this plasmid. *Mol. Gen. Genet.* 210 101–110. 10.1007/bf00337764 3323833

[B4] Cameron E. A. Curtis M. M. Kumar A. Dunny G. M. Sperandio V. (2018). Microbiota and pathogen proteases modulate type III secretion activity in Enterohemorrhagic *Escherichia coli*. *mBio* 9 e02204–18. 10.1128/mBio.02204-18 30514785 PMC6282197

[B5] Cheverton A. M. Gollan B. Przydacz M. Wong C. T. Mylona A. Hare S. A. (2016). A *Salmonella* toxin promotes persister formation through acetylation of tRNA. *Mol. Cell* 63 86–96. 10.1016/j.molcel.2016.05.002 27264868 PMC4942678

[B6] Eshun-Wilson L. Zhang R. Portran D. Nachury M. V. Toso D. B. Löhr T. (2019). Effects of α-tubulin acetylation on microtubule structure and stability. *Proc. Natl. Acad. Sci. U.S.A.* 116 10366–10371. 10.1073/pnas.1900441116 31072936 PMC6535015

[B7] Fraikin N. Goormaghtigh F. Van Melderen L. (2020). Type II toxin-antitoxin systems: evolution and revolutions. *J. Bacteriol*. 202 e00763–19. 10.1128/JB.e00763-19 31932311 PMC7167474

[B8] Gerdes K. Christensen S. K. Lobner-Olesen A. (2005). Prokaryotic toxin-antitoxin stress response loci. *Nat. Rev. Microbiol.* 3 371–382. 10.1038/nrmicro1147 15864262

[B9] Gerdes K. Rasmussen P. B. Molin S. (1986). Unique type of plasmid maintenance function: postsegregational killing of plasmid-free cells. *Proc. Natl. Acad. Sci. U.S.A.* 83 3116–3120. 10.1073/pnas.83.10.3116 3517851 PMC323463

[B10] Goormaghtigh F. Fraikin N. Putrins M. Hauryliuk V. Garcia-Pino A. Udekwu K. (2018a). Reply to holden and errington, “Type II toxin-antitoxin systems and persister cells”. *mBio* 9:e01838–18. 10.1128/mBio.01838-18 30254127 PMC6156200

[B11] Goormaghtigh F. Fraikin N. Putrinš M. Hallaert T. Hauryliuk V. Garcia-Pino A. (2018b). Reassessing the role of type II toxin-antitoxin systems in formation of *Escherichia coli* type II persister cells. *mBio* 9:e00640–18. 10.1128/mbio.00640-18 29895634 PMC6016239

[B12] Harms A. Brodersen D. E. Mitarai N. Gerdes K. (2018). Toxins, targets, and triggers: an overview of toxin-antitoxin biology. *Mol. Cell* 70 768–784. 10.1016/j.molcel.2018.01.003 29398446

[B13] Holden D. W. Errington J. (2018). Type II toxin-antitoxin systems and persister cells. *mBio* 9 e01574–18. 10.1128/mBio.01574-18 30254124 PMC6156201

[B14] Hosseini N. Pourhajibagher M. Chiniforush N. Hosseinkhan N. Rezaie P. Bahador A. (2019). Modulation of toxin-antitoxin system Rnl AB Type II in phage-resistant Gammaproteobacteria surviving photodynamic treatment. *J. Lasers Med. Sci.* 10 21–28. 10.15171/jlms.2019.03 31360364 PMC6499577

[B15] Jurenas D. Chatterjee S. Konijnenberg A. Sobott F. Droogmans L. Garcia-Pino A. (2017). AtaT blocks translation initiation by N-acetylation of the initiator tRNA(fMet). *Nat. Chem. Biol.* 13 640–646. 10.1038/nchembio.2346 28369041

[B16] Jurënas D. Van Melderen L. (2020). The variety in the common theme of translation inhibition by Type II toxin-antitoxin systems. *Front. Genet.* 11:262. 10.3389/fgene.2020.00262 PMC7180214 32362907

[B17] Lang C. Fruth A. Holland G. Laue M. Muhlen S. Dersch P. (2018). Novel type of pilus associated with a Shiga-toxigenic *E. coli* hybrid pathovar conveys aggregative adherence and bacterial virulence. *Emerg. Microbes Infect.* 7:203. 10.1038/s41426-018-0209-8 30514915 PMC6279748

[B18] Li T. Li Z. Chen F. Liu X. Ning N. Huang J. (2017). Eukaryotic-like kinase expression in enterohemorrhagic *Escherichia coli*: potential for enhancing host aggressive inflammatory response. *J. Infect. Dis.* 216 1150–1158. 10.1093/infdis/jix160 29186483

[B19] Lobato-Marquez D. Diaz-Orejas R. Garcia-Del Portillo F. (2016). Toxin-antitoxins and bacterial virulence. *FEMS Microbiol. Rev.* 40 592–609. 10.1093/femsre/fuw022 27476076

[B20] Luo W. Donnenberg M. S. (2006). Analysis of the function of enteropathogenic *Escherichia coli* EspB by random mutagenesis. *Infect. Immun.* 74 810–820. 10.1128/iai.74.2.810-820.2006 16428723 PMC1360311

[B21] Luo W. Donnenberg M. S. (2011). Interactions and predicted host membrane topology of the enteropathogenic *Escherichia coli* translocator protein EspB. *J. Bacteriol.* 193 2972–2980. 10.1128/jb.00153-11 21498649 PMC3133209

[B22] McVicker G. Tang C. M. (2016). Deletion of toxin-antitoxin systems in the evolution of *Shigella* sonnei as a host-adapted pathogen. *Nat. Microbiol.* 2:16204. 10.1038/nmicrobiol.2016.204 27819667

[B23] McWilliams B. D. Torres A. G. (2014). Enterohemorrhagic *Escherichia coli* adhesins. *Microbiol. Spectr.* 2:3. 10.1128/microbiolspec.EHEC-0003-2013 26103974

[B24] Mills E. Baruch K. Aviv G. Nitzan M. Rosenshine I. (2013). Dynamics of the type III secretion system activity of enteropathogenic *Escherichia coli*. *mBio* 4 e00303–13. 10.1128/mBio.00303-13 23900171 PMC3735188

[B25] Ogura T. Hiraga S. (1983). Mini-F plasmid genes that couple host cell division to plasmid proliferation. *Proc. Natl. Acad. Sci. U.S.A.* 80 4784–4788. 10.1073/pnas.80.15.4784 6308648 PMC384129

[B26] Paul P. Sahu B. R. Suar M. (2019). Plausible role of bacterial toxin-antitoxin system in persister cell formation and elimination. *Mol Oral Microbiol* 34 97–107. 10.1111/omi.12258 30891951

[B27] Pontes M. H. Groisman E. A. (2019). Slow growth determines nonheritable antibiotic resistance in *Salmonella enterica*. *Sci Signal* 12:eaax3938. 10.1126/scisignal.aax3938 31363068 PMC7206539

[B28] Qian H. Yao Q. Tai C. Deng Z. Gan J. Ou H. Y. (2018). Identification and characterization of acetyltransferase-type toxin-antitoxin locus in *Klebsiella pneumoniae*. *Mol. Microbiol.* 108 336–349. 10.1111/mmi.13934 29461656

[B29] Qian H. Yu H. Li P. Zhu E. Yao Q. Tai C. (2019). Toxin-antitoxin operon kacAT of *Klebsiella pneumoniae* is regulated by conditional cooperativity via a W-shaped KacA-KacT complex. *Nucleic Acids Res.* 47 7690–7702. 10.1093/nar/gkz563 31260525 PMC6698736

[B30] Ren J. Sang Y. Tan Y. Tao J. Ni J. Liu S. (2016). Acetylation of lysine 201 inhibits the DNA-binding ability of PhoP to regulate *Salmonella* virulence. *PLoS Pathog.* 12:e1005458. 10.1371/journal.ppat.1005458 26943369 PMC4778762

[B31] Riffaud C. Pinel-Marie M. L. Felden B. (2020). Cross-Regulations between bacterial toxin-antitoxin systems: evidence of an interconnected regulatory network? *Trends Microbiol*. 28 851–866. 10.1016/j.tim.2020.05.016 32540313

[B32] Rocker A. Meinhart A. (2016). Type II toxin: antitoxin systems. More than small selfish entities? *Curr. Genet.* 62 287–290. 10.1007/s00294-015-0541-7 26597447 PMC4826407

[B33] Rosendahl S. Tamman H. Brauer A. Remm M. Horak R. (2020). Chromosomal toxin-antitoxin systems in *Pseudomonas* putida are rather selfish than beneficial. *Sci. Rep.* 10:9230. 10.1038/s41598-020-65504-0 PMC7280312 32513960

[B34] Rycroft J. A. Gollan B. Grabe G. J. Hall A. Cheverton A. M. Larrouy-Maumus G. (2018). Activity of acetyltransferase toxins involved in *Salmonella* persister formation during macrophage infection. *Nat. Commun.* 9:1993. 10.1038/s41467-018-04472-6 29777131 PMC5959882

[B35] Sang Y. Ren J. Ni J. Tao J. Lu J. Yao Y. F. (2016). Protein acetylation is involved in *Salmonella enterica* serovar typhimurium virulence. *J. Infect. Dis.* 213 1836–1845. 10.1093/infdis/jiw028 26810370

[B36] Sauert M. Wolfinger M. T. Vesper O. Muller C. Byrgazov K. Moll I. (2016). The MazF-regulon: a toolbox for the post-transcriptional stress response in *Escherichia coli*. *Nucleic Acids Res.* 44 6660–6675. 10.1093/nar/gkw115 26908653 PMC5001579

[B37] Song S. Wood T. K. (2020). Toxin/Antitoxin system paradigms: toxins bound to antitoxins are not likely activated by preferential antitoxin degradation. *Adv. Biosyst.* 4:e1900290. 10.1002/adbi.201900290 32293143

[B38] Song T. Su H. Yin W. Wang L. Huang R. (2019). Acetylation modulates LC3 stability and cargo recognition. *FEBS Lett.* 593 414–422. 10.1002/1873-3468.13327 30633346

[B39] Tsilibaris V. Maenhaut-Michel G. Mine N. Van Melderen L. (2007). What is the benefit to *Escherichia coli* of having multiple toxin-antitoxin systems in its genome? *J. Bacteriol.* 189 6101–6108. 10.1128/JB.00527-07 17513477 PMC1951899

[B40] Van Melderen L. Jurenas D. Garcia-Pino A. (2018). Messing up translation from the start: how AtaT inhibits translation initiation in *E. coli*. *RNA Biol.* 15 303–307. 10.1080/15476286.2017.1391439 29099338 PMC5927719

[B41] Van Melderen L. Saavedra De Bast M. (2009). Bacterial toxin-antitoxin systems: more than selfish entities? *PLoS Genet.* 5:e1000437. 10.1371/journal.pgen.1000437 19325885 PMC2654758

[B42] VanDrisse C. M. Parks A. R. Escalante-Semerena J. C. (2017). A toxin involved in *Salmonella* persistence regulates its activity by acetylating its cognate antitoxin, a modification reversed by CobB sirtuin deacetylase. *mBio* 8 e00708–17. 10.1128/mBio.00708-17 28559487 PMC5449658

[B43] Walling L. R. Butler J. S. (2019). Toxins targeting transfer RNAs: translation inhibition by bacterial toxin-antitoxin systems. *Wiley Interdiscip. Rev. RNA* 10:e1506. 10.1002/wrna.1506 30296016 PMC6294680

[B44] Wen Y. Behiels E. Devreese B. (2014). Toxin-Antitoxin systems: their role in persistence, biofilm formation, and pathogenicity. *Pathog. Dis.* 70 240–249. 10.1111/2049-632X.12145 24478112

[B45] Wilcox B. Osterman I. Serebryakova M. Lukyanov D. Komarova E. Gollan B. (2018). *Escherichia coli* ItaT is a type II toxin that inhibits translation by acetylating isoleucyl-tRNAIle. *Nucleic Acids Res.* 46 7873–7885. 10.1093/nar/gky560 29931259 PMC6125619

[B46] Xie Y. Wei Y. Shen Y. Li X. Zhou H. Tai C. (2018). TADB 2.0: an updated database of bacterial type II toxin-antitoxin loci. *Nucleic Acids Res.* 46 D749–D753. 10.1093/nar/gkx1033 29106666 PMC5753263

[B47] Yamaguchi Y. Inouye M. (2011). Regulation of growth and death in *Escherichia coli* by toxin-antitoxin systems. *Nat. Rev. Microbiol.* 9 779–790. 10.1038/nrmicro2651 21927020

[B48] Yashiro Y. Yamashita S. Tomita K. (2019). Crystal structure of the enterohemorrhagic *Escherichia coli* AtaT-AtaR Toxin-antitoxin complex. *Structure* 27 476–484 e473. 10.1016/j.str.2018.11.005 30612860

[B49] Yeo C. C. (2018). GNAT toxins of bacterial toxin-antitoxin systems: acetylation of charged tRNAs to inhibit translation. *Mol. Microbiol.* 108 331–335. 10.1111/mmi.13958 29624768

