# Supplementary material for: AtaT Improves the Stability of Pore-Forming Protein EspB by Acetylating Lysine 206 to Enhance Strain Virulence
Source: Front Microbiol. 2021 Mar 1;12:627141. doi: 10.3389/fmicb.2021.627141 (PMC7957018; doi:10.3389/fmicb.2021.627141)
Supplement: Supplementary file 1 [file Data_Sheet_1.pdf]

Supplementary Materials

Supplementary Table1： Strains used in this study

Strain	Description	Source or reference
EHEC WT	Wild-type EHEC serotype O157:H7 EDL933	[1]
Δ*ataT*	EHEC WT Δ*ataT*	This study
Δ*ataR*	EHEC WT Δ*ataR*	This study
EspB/WT	Plasmid pETDuet-1-EspB(his)$_6$ cloned into EHEC WT	This study
EspB/Δ*ataT*	Plasmid pETDuet-1-EspB(his)$_6$ cloned into Δ*ataT*	This study
EspB/Δ*ataR*	Plasmid pETDuet-1-EspB(his)$_6$ cloned into Δ*ataR*	This study
EspBK206A/EHEC	Plasmid pETDuet-1-EspBK206A(his)$_6$ cloned into EHEC WT	This study
EspBK206Q/EHEC	Plasmid pETDuet-1-EspBK206Q(his)$_6$ cloned into EHEC WT	This study
EspBK206R/EHEC	Plasmid pETDuet-1-EspBK206R(his)$_6$ cloned into EHEC WT	This study
TEM1/WT	Plasmid pETDuet-1-TEM1 cloned into EHEC WT	This study
Tir-TEM1/WT	Plasmid pETDuet-1-Tir-TEM1 cloned into EHEC WT	This study
Tir-TEM1/Δ*ataT*	Plasmid pETDuet-1-Tir-TEM1 cloned into Δ*ataT*	This study
Tir-TEM1/Δ*ataR*	Plasmid pETDuet-1-Tir-TEM1 cloned into Δ*ataR*	This study

BL21(DE3)	*E. coli* engineering bacteria	
pET-EspA/B21	BL21(DE3) containing plasmid pETDuet-1-EspA(his)$_6$	This study
pET-EspA-pBAD-T/B21	BL21(DE3) containing both plasmid pETDuet-1-EspA(his)$_6$ and pBAD-AtaT	This study
pET-EspB/B21	BL21(DE3) containing plasmid pETDuet-1-EspB(his)$_6$	This study
pET-EspB-pBAD-T/B21	BL21(DE3) containing both plasmid pETDuet-1-EspB(his)$_6$ and pBAD-AtaT	This study
pET-LpfA/B21	BL21(DE3) containing plasmid pETDuet-1-LpfA(his)$_6$	This study
pET-LpfA-pBAD-T/B21	BL21(DE3) containing both plasmid pETDuet-1-LpfA(his)$_6$ and pBAD-AtaT	This study
pET-Tccp/B21	BL21(DE3) containing plasmid pETDuet-1-Tccp(his)$_6$	This study
pET-Tccp-pBAD-T/B21	BL21(DE3) containing both plasmid pETDuet-1-Tccp(his)$_6$ and pBAD-AtaT	This study
pET-Intim/B21	BL21(DE3) containing plasmid pETDuet-1-Intim(his)$_6$	This study
pET-Intim-pBAD-T/B21	BL21(DE3) containing both plasmid pETDuet-1-Intim(his)$_6$ and pBAD-AtaT	This study
pET-Tir/B21	BL21(DE3) containing plasmid pETDuet-1-Tir(his)$_6$	This study
pET-Tir-pBAD-T/B21	BL21(DE3) containing both plasmid pETDuet-1-Tir(his)$_6$ and pBAD-AtaT	This study

Supplementary Table2：Plasmids used in this study

Plasmids	Description	Source or reference
pUC19-T-UKD	Plasmid containing kanamycin-resistant gene kan (flanked by FRT sites) flanked by homologous of *ataT*	This study
pUC19-A-UKD	Plasmid containing kanamycin-resistant gene kan (flanked by FRT sites) flanked by homologous of *ataR*	This study
pKD46	Plasmid uses the pBAD promoter to express λ-Red recombinase from a low copy number temperature-sensitive replicon.	[2]
pFLP2	Plasmid was used to flip out the marker gene used for mutagenesis.	[3]
pBAD-T	pBAD33-containing *ataT*	This study
pET-AT$(his)_6$	pETDuet-1 containing *ataRT* with 6 his tag in the N termini of *ataT*	This study
pET-EspA$(his)_6$	pETDuet-1 containing *espA* with 6 his tag	This study
pET-EspB$(his)_6$	pETDuet-1 containing *espB* with 6 his tag	This study
pET-LpfA$(his)_6$	pETDuet-1 containing *lpfA* with 6 his tag	This study
pET-TccP$(his)_6$	pETDuet-1 containing *tccp* with 6 his tag	This study

pET-Intim (his)$_6$	pETDuet-1 containing *eae* with 6 his tag	This study
pET-Tir (his)$_6$	pETDuet-1 containing *tir* with 6 his tag	This study
pET-Tir -TEM1	pETDuet-1 containing *tir* with β-lactamase TEM-1	This study
pET-TEM1	pETDuet-1 containing β-lactamase TEM-1	This study
pET-BK206A(his)$_6$	pETDuet-1 containing *espB*K206A with with 6 his tag	This study
pET-BK206Q(his)$_6$	pETDuet-1 containing *espB*K206Q with with 6 his tag	This study
pET-BK206R(his)$_6$	pETDuet-1 containing *espB*K206R with with 6 his tag	This study

Supplementary Table 3：Acetylation sites of EspB in vivo identified by LC-MS/MS analyses.

Acetylation sites*	Peptide sequence	Number of acetylated peptides /total number of the same peptides	
		Untreated	AtaT treated
K47	VDICK*LMLEIQK	0/0	1/2
K54	LMLEIQK*LLGK	2/3	2/5
K58	LLGK*MVTLLQDYQQK	0/1	1/3
K92	AIEEK*K	0/1	1/2
K131	GAGEIAEK*ASSASSK	1/5	1/4
K138	ASSASSK*AAGAASEVANK	1/3	2/5
K149	AAGAASEVANK*ALVK	2/7	2/9
K178	AMATTTK*AASR	0/4	1/9
K192	ASGVADDVAK*ASDFAEDLADAAEK	0/1	2/3
K206	ASDFAEDLADAAEK*TSR	0/3	3/3

Notes: LC, liquid chromatography–tandem; MS, mass spectroscopy.

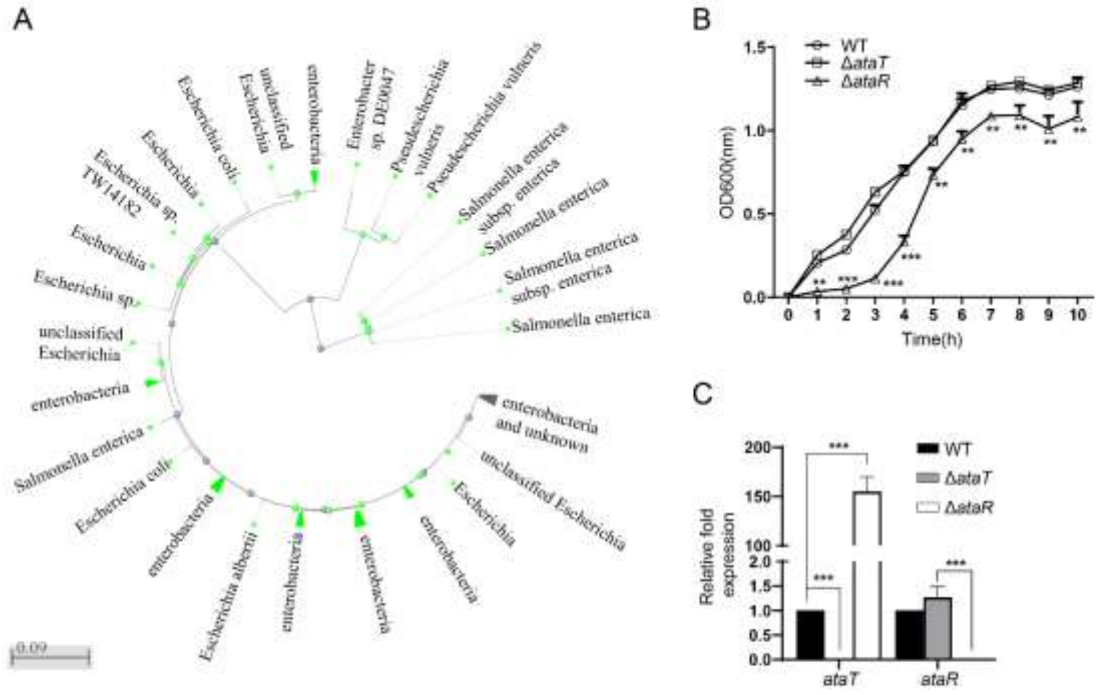

Supplementary Figure 1. (A) AtaRT TA molecules are mainly distributed in pathogenic bacteria (*Escherichia*, *Shigella*, *Klebsiella*, *Salmonella*, et al.) and are distributed in pairs. Distance tree was assigned by NCBI, protein identify>=75%; **(B)** Growth curves of EHEC WT, Δ*ataT,* and Δ*ataR* strains. The mRNA expression of ataRT showed typical TA molecular characteristics as **(C)** , *ataT* gene overexpressed after *ataR* deleted, and the strain showed growth inhibition. Data are represented as mean ± SEM, ** P < 0.01,***P<0.001. ALL experiments shown were repeated at least two times. Abbreviations: NCBI, National Center for Biotechnology Information; WT, wild-type.

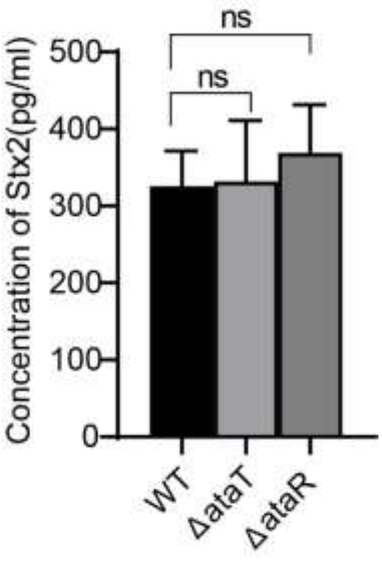

Supplementary Figure 2. Stx2 level of different strains. HT-29 cells were infected with EHEC WT and mutants for 6h. The supernatant mixtures were collected, and the Stx2 levels were determined by ELISA. Data are represented as mean ± SEM.

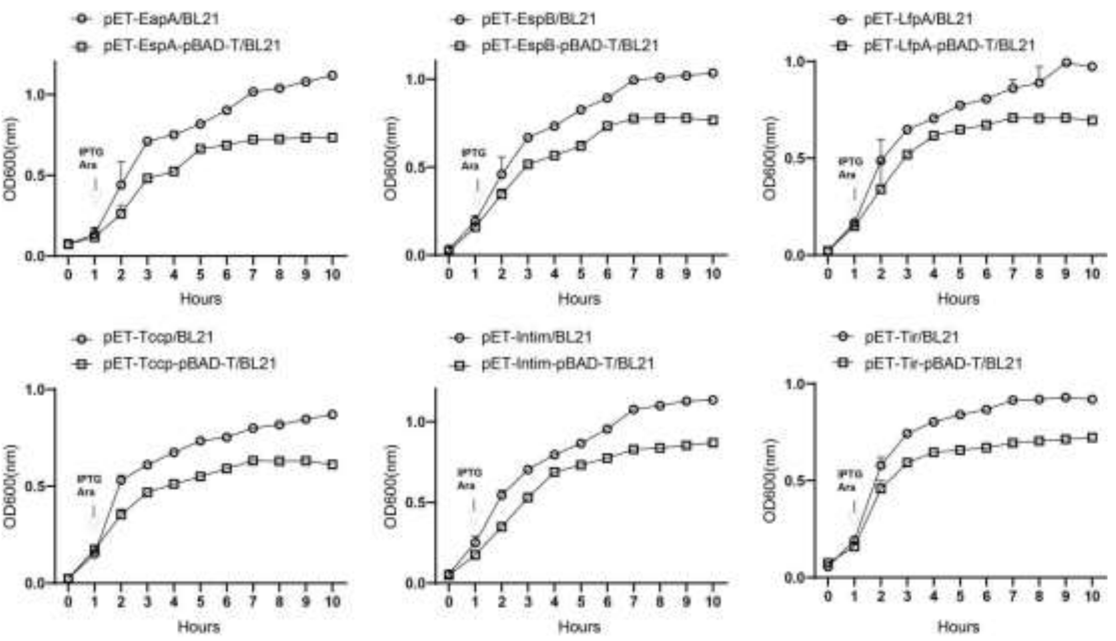

Supplementary Figure 3. Growth curves of adhesion proteins in *E. coli* strain BL21 with or without AtaT expression. The adhesion proteins were expressed using pETDuet-1, and pBAD33 expressed AtaT. Strains harboring plasmids were induced by 10mM IPTG, and 0. 2% arabinose simultaneously. Abbreviations: IPTG, isopropyl-β-D-thiogalactopyranoside

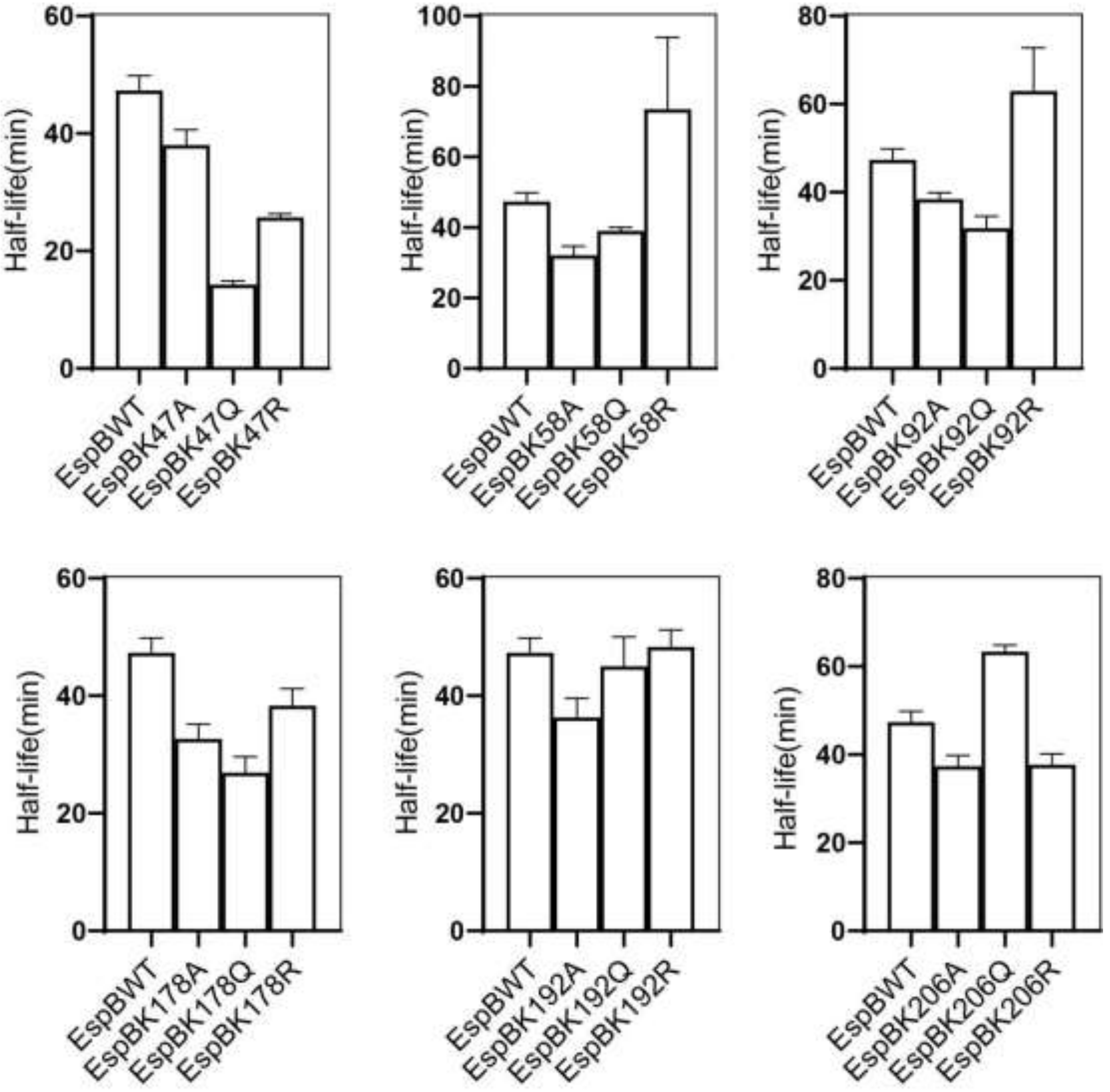

Supplementary Figure 4. Stabilities of EspB mutants and wild type in EHEC. Strains harboring EspB expression plasmid was induced by IPTG for 60 minutes. The translation was terminated with spectinomycin, and samples were collected at different time points for Western blot analysis. The level of EspB was determined by the anti-His antibody, and the half-life were calculated by the gray value. Experiments shown were repeated at least three times, and data are represented as mean ± SEM.

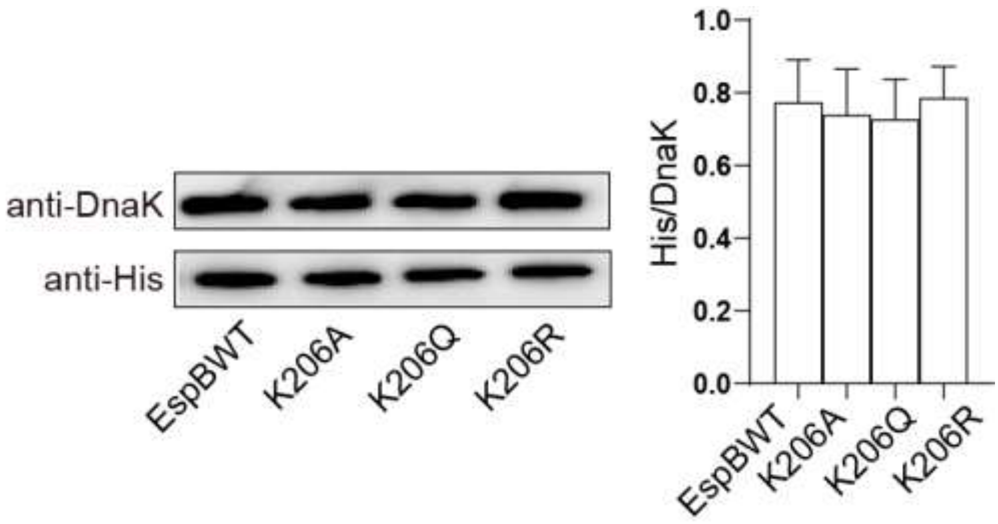

Supplementary Figure 5. Levels of EspB mutants and wild type in EHEC. Strains harboring EspB expression plasmid was induced by IPTG. The samples were collected at OD600 nm= 0.6 for Western blot analysis. The level of EspB was determined by the anti-His antibody, DnaK used as a control. The level of EspB were calculated by the His/DnaK gray ratio. Experiments shown were repeated at least three times, and data are represented as mean ± SEM. WT, wild type.

Supplementary References

[1]. Li T, Li Z, Chen F, et al. Eukaryotic-like Kinase Expression in Enterohemorrhagic Escherichia coli: Potential for Enhancing Host Aggressive Inflammatory Response. J Infect Dis **2017**; 216:1150-8.

[2]. Datsenko KA, Wanner BL. One-step inactivation of chromosomal genes in Escherichia coli K-12 using PCR products. Proceedings of the National Academy of Sciences of the United States of America **2000**; 97:6640-5.

[3]. Bi D, Jiang X, Sheng ZK, et al. Mapping the resistance-associated mobilome of a carbapenem-resistant Klebsiella pneumoniae strain reveals insights into factors shaping these regions and facilitates generation of a 'resistance-disarmed' model organism. J Antimicrob Chemother **2015**; 70:2770-4.
